# Time-Driven Effects on Processing Relative Clauses

**DOI:** 10.1007/s10936-015-9391-1

**Published:** 2015-09-04

**Authors:** Andrea Schremm, Merle Horne, Mikael Roll

**Affiliations:** Department of Linguistics and Phonetics, Centre for Languages and Literature, Lund University, Box 201, 221 00 Lund, Sweden

**Keywords:** Sentence processing, Response times, Agreement, Semantic congruency

## Abstract

The present response time study investigated how a hypothesized time-based working memory constraint of 2–3 s affects the resolution of grammatical and semantic dependencies. Congruent and incongruent object relative (OR) and subject relative sentences were read at different presentation rates so that the distance between dependent words was either shorter or longer than 2–3 s. Incongruent OR sentences showed an effect of presentation rate. Experiment 1 focused on grammatical dependencies. Processing of adjectives with agreement features mismatching those of the preceding dependent word showed rapid agreement resolution at a time-interval below 2 s. Dependency intervals over 3 s reflected a different, more time-consuming process possibly due to extended search in sentence semantic representations as the grammatical form of the first word in the dependency fades away. In experiment 2, focusing on semantic dependencies, incongruent OR sentences displayed a different pattern: a gradual increase in processing time as a function of distance between dependent words. Thus, the 2–3 s long time-window seems to constrain the maintenance of grammatical forms in working memory.

## Introduction

It is a well-established finding that sentences containing object relative (OR) clauses are generally more difficult to comprehend than corresponding sentences containing subject relative (SR) clauses (Gordon et al. 2001, 2004; King and Just 1991; Kwon et al. 2013; Schriefers et al. 1995; Traxler et al. 2002). In a sentence containing an object relative (OR) clause such as example (1), the relative pronoun *that *functions as the object of the subordinate verb (*sings*), while in the subject relative counterpart in example (2), *that* is interpreted as the subject of the verb *sings*.

1. It is the psalm that the choir always sings (OR).
2. It is the choir that always sings the psalm (SR).

A number of factors have been argued to contribute to the observed complexity difference. One of these is the greater frequency with which language users encounter the simple subject–verb–object word order characterizing SRs, as opposed to the non-canonical word order in OR sentences (e.g. MacDonald and Christiansen 2002). Another factor is the interference between two noun phrases (e.g. *psalm* and *choir* in (1)) that need to be integrated with the subordinate verb (e.g. *sings* in (1)) in ORs (Gordon et al. 2001). Other accounts attribute the processing complexity to an increase in working memory load (e.g. Gibson 1998; Lewis and Vasishth 2005; Nakatani and Gibson 2010). The very distance between dependent elements such as the extraposed object (*the psalm*) and the relative clause verb (*sings*) is often longer in OR sentences than in SR sentences, a fact which might increase working memory demands due to variation in the number and type of intervening items denoting new discourse referents (Gibson 1998, 2000; Warren and Gibson 2002). Furthermore, interference effects might arise as a larger number of sentence constituents are processed before the dependency is completely resolved. This could affect the success with which a preceding noun phrase (NP) object could be retrieved at the subordinate clause verb in OR sentences (Lewis and Vasishth 2005; Vasishth and Lewis 2006). Importantly, the temporal interval that separates the dependent elements in OR sentences is also greater than that in corresponding SR sentences.

Results from studies on grammatical and prosodic processing indicate that a time-window of 2–3 s constrains the integration of formal (e.g. phonological, morphological and syntactic) information in sentence processing (Roll et al. 2013, 2012; Vollrath et al. 1992). These results are also in line with research in other areas of investigation. Baddeley (1997), in his work on human memory, has claimed that the part of working memory where speech processing takes place (‘inner speech’) has a time limit of around 2 s. As linguistic dependencies in OR sentences may span intervals that take longer than 2–3 s to process, the proposed time limit on working memory could contribute to the observed processing difficulty associated with these structures. Thus, our goal in the present study was to investigate, in two response time experiments, how the integration of grammatical features and semantic information in dependency resolution were influenced when the temporal distance separating dependent words in Swedish OR and SR sentences was either shorter or longer than the assumed time-window of 2–3 s.

## Time Constraints on Verbal Working Memory

Sachs (1974) found that, already after a 4 s long delay, participants reading short texts were unable to reliably recognize changes that concerned the exact wording and the form of the sentences. Alterations involving meaning were, however, successfully identified even after 23 s, suggesting that after a brief period of only a few seconds, rapidly fading form-based cues are recoded into more long-lasting semantic (propositional) representations. These findings are indicative of a short temporal interval for the integration of grammatical information in language processing, which might be the manifestation of a more general neurocognitive principle. Pöppel (1997) has suggested that the processing of sensory information is characterized by an integration mechanism that binds sequences of events perceived arriving within a time-window of 3 s into units.

The decay of grammatical information within 3 s has also been shown to affect the processing of agreement dependencies spanning different time-intervals. In an event-related potentials (ERP) study, Roll et al. (2013) recorded participants’ brain responses as they read Swedish sentences in which the adjective (e.g. *snäll* ‘kind-sg’) agreed or disagreed in number with the personal pronoun (e.g. *vi *‘we-pl’) it modified. If the temporal distance between the disagreeing words was shorter than 2.5 s, the mismatch yielded an ERP signal (left-lateralized negativity) commonly associated with the detection of morphosyntactic violations. The violation was thus processed as a mismatch between grammatical forms, indicating that the form-based representations of both words in the dependency were still present in short-term memory. When the distance exceeded 3 s, the ERP response elicited by a disagreeing adjective changed, suggesting the involvement of different agreement-processing mechanisms. The obtained brain response had a later onset and was characterized by a shift in spatial distribution from left to right-hemisphere. It seems that as the grammatical form of the pronoun faded in short-term memory, agreement-resolution required a more time-consuming extended search in order to retrieve the item that the adjective modified. The observed distributional change towards right-lateralization might also be indicative of the role of propositional semantic content, as opposed to grammatical features, in agreement resolution at intervals beyond 3 s.

Similar to the findings in Sachs (1974), these results indicate that the 2–3 s long working memory constraint applies to the retention of grammatical forms, constituting a time-window during which decaying linguistic information is integrated into longer-lasting and more abstract propositional semantic representations. However, although different brain responses have been found for intervals over and under 3 s, it is still unknown whether the temporal distance affects behavioral measures.

## Current Study

Using response time measures, we investigated how time constraints on working memory influence the processing of sentences containing ORs and SRs. The temporal interval between dependent words was manipulated by varying the speed with which sentences were read, while other factors that have been proposed to contribute to the processing difficulty of ORs were kept constant. As previous results suggest that grammatical forms versus semantic representations may decay in memory at different rates, two experiments were conducted. The grammatical congruency experiment tested the resolution of dependencies between grammatically matching versus mismatching word forms, whereas the semantic congruency experiment focused on the integration of semantically congruent versus incongruent constituents. The presentation rate of stimulus sentences in both experiments was varied so that the distance between the dependent elements in OR sentences (such as between *fönstret *‘the-window’ and *öppet *‘open’ in sentence (5) of Table 1 and between *bröd *‘bread’ and *bakar *‘bakes’ in sentence (11) of Table 2) was within the assumed 3 s window at fast word presentation rate (1759 ms), slightly exceeded the window at medium presentation rate (3375 ms) and was well beyond the 3 s window at slow rate (5250 ms). In the SR sentences, the dependent elements were adjacent and always appeared within the hypothesized time-window regardless of the presentation rate.

**Table 1:** Example stimuli from the grammatical congruency experiment

Condition		Example sentence
SR, grammatical	(1)	Det är David som gärna har fönstret öppet
		‘It is David that gladly has the-window open-SG’
	(2)	Det är David som gärna har fönstren öppna
		‘It is David that gladly has the-windows open-PL’
SR, ungrammatical	(3)	* Det är David som gärna har fönstret öppna
		‘It is David that gladly has the-window open-PL’
	(4)	* Det är David som gärna har fönstren öppet
		‘It is David that gladly has the-windows open-SG’
OR, grammatical	(5)	Det är fönstret som David gärna har öppet
		‘It is the-window that David gladly has open-SG’
	(6)	Det är fönstren som David gärna har öppna
		‘It is the-windows that David gladly has open-PL’
OR, ungrammatical	(7)	* Det är fönstret som David gärna har öppna
		‘It is the-window that David gladly has open-PL’
	(8)	* Det är fönstren som David gärna har öppet
		‘It is the-windows that David gladly has open-SG’

*SR* subject relative sentence, *OR* object relative sentence

**Table 2:** Example stimuli from the semantic congruency experiment

Condition		Example sentence
SR, congruent	(9)	Det är flickan som ganska ofta bakar bröd
		‘It is the-girl that quite often bakes bread’
SR, incongruent	(10)	* Det är flickan som ganska ofta läser bröd
		‘It is the-girl that quite often reads bread’
OR, congruent	(11)	Det är bröd som flickan ganska ofta bakar
		‘It is bread that the-girl quite often bakes’
OR, incongruent	(12)	* Det är bröd som flickan ganska ofta läser
		‘It is bread that the-girl quite often reads’

*SR* subject relative sentence, *OR* object relative sentence

The sentences of the grammatical congruency experiment (Table 1) involved an agreement dependency between a NP (*fönstr-et/-en* ‘the window-sg/pl’) and a following adjective (*öpp-et/-na* ‘open-sg/pl’). Participants’ response times were measured at the adjective, where they were required to judge if the sentence-final word was correct or incorrect. In examples (1)–(2) and (5)–(6) of Table 1, the adjective agrees with the preceding NP in number: it takes the suffix –*et *when it appears with a singular NP, and the suffix -*na *when used together with a plural NP.

At fast presentation rate, where the adjective and the antecedent NP appear within the same time-window in both OR and SR sentences, the grammatical form of the NP would be expected to still be activated upon reading the adjective (Roll et al. 2013, 2012). Consequently, the (mis)matching item would be found in short-term memory and its number features could be rapidly checked against the form of the adjective. At medium and slow presentation rates, the temporal distance between the dependent elements in OR sentences exceeds the hypothesized processing window. Therefore, the grammatical form of the NP would be expected to have vanished from working memory, resulting in extended search in memory involving sentence semantic (propositional) information and thus longer decision times at the adjective.

It could be thought that there might be a processing difference between sentences ending in agreeing as opposed to disagreeing adjectives. Upon encountering the final word (=adjective) in an agreeing dependency sentence, readers might be able to judge the appropriateness of the adjective based on its fit in the emerging overall sentence representation available at that point (e.g*. the windows... open-pl*). To be able to make a decision, therefore, no extensive memory search would be expected for the previously presented member of the dependency. In examples (3)–(4) and (7)–(8), however, the adjective fails to agree with the appropriate preceding NP. After reading a disagreeing adjective, decision times could be thought to reflect the latency of the agreement dependency-resolution process triggered by the suffix expressing number. This process would be expected to involve a search in short-term memory for an appropriate grammatical form that the adjective could agree with, i.e. an NP with singular or plural marking.

The semantic congruency experiment (Table 2) focused on the semantic congruency between the object of the relative clause (*bröd *‘bread’) and the verb it is integrated with (*bakar/läser *‘bakes’/‘reads’).^1^ Response times were recorded for the sentence-final word, which was either semantically congruent as in sentences (9) and (11) in Table 2 or semantically incongruent as in (10) and (12). Encountering a semantically incongruent final word such as the verb *läser *‘reads’ following the noun *bröd *‘bread’ in (12), readers could be thought to re-evaluate semantic information from the previously processed NP in order to judge its suitability in the semantic dependency. As sentence meaning representations have been found to be maintained in memory for longer time-periods (Sachs 1974), resolution of the semantic congruency between the verb and the extraposed object NP might engage similar processes at all three presentation rates, even when the distance between the semantically dependent words exceeds the 2–3 s time-window. Therefore, the semantic congruency task was predicted to produce different response time patterns than the syntactic task involving decisions on agreement relations: instead of indicating a sharp dividing line between results at fast presentation rate on the one hand and medium and slow rates on the other hand, response latencies in the semantic congruency task might reflect either a more gradual change as the temporal distance between the dependent words increases and word meaning representations fade or no measurable effect of presentation rate if word meaning representations are maintained, at least to some degree, in sentence meaning representations.

## Method

### Participants

In the grammatical congruency experiment, 28 native speakers of Swedish, 16 women and 12 men, participated. Mean age was 24.4 years, *SD* = 3.27. In the semantic congruency experiment, 28 Swedish native speakers participated; none had taken part in the grammatical congruency experiment. Seventeen of the participants were women and eleven were men. Mean age was 25.9 years, *SD* = 4.90.

### Materials

Both experiments involved 40 OR and 40 SR sentences in Swedish. Sample sentences for each test condition are shown in Table 1 for the grammatical congruency experiment and in Table 2 for the semantic congruency experiment. The OR and SR sentences were created as each other’s counterparts, containing the same words. The 80 test-sentences were presented at three different rates, resulting in a total of 240 trials per experiment. The sentences consisted of eight monosyllabic or disyllabic words and had the following structure: *Det* ‘it’ + copular verb *är *‘is/are’ + NP + relative clause. The clefted NP following the copular verb (*är *‘is/are’) was the object of the relative clause verb in the OR sentences and the subject of the relative clause verb in the SR sentences. The relative clause was introduced by the relative pronoun *som *‘that’, which may refer to both human and inanimate nouns.

*Grammatical congruency experiment.* In the grammatical congruency experiment, each sentence contained an inanimate definite NP (*fönstret *‘the-window’ or *fönstren *‘the-windows’) and a sentence-final adjective that was in a number agreement relation with the NP (*öppet *‘open-sg’, *öppna *‘open-pl’). The two constituents formed an object predicative construction (e.g. *fönstret öppet *‘the-window open-sg’), in which the adjective (*öppet *‘open-sg’) functioned as the predicate phrase, modifying the object complement (*fönstret *‘the-window’) of the relative clause verb (*har *‘has’). Thus, in the SR sentences, the sentence-final adjective directly followed the NP it modified. In the OR sentences where the same NP appeared as the clefted object, i.e. the non-subject complement of the copular verb (*är *‘is/are’) in the matrix clause, the object NP and the adjective were separated by 4 intervening words.

Ten different noun–adjective pairs were used. All the nouns had the same grammatical gender (neuter), a regular singular definite form expressed by the definite article –*et* (*fönstret *‘the-window’) and a definite plural formed by the suffix –*en *(*fönstren *‘the-windows’). Each noun and adjective appeared in both singular and plural forms, resulting in two grammatically correct and two grammatically incorrect combinations for each pair: singular noun–singular adjective (*fönstret *– *öppet*), plural noun–plural adjective (*fönstren *–*öppna*), singular noun–plural adjective (*fönstret *– *öppna*, mismatch), plural noun–singular adjective (*fönstren *–*öppet*, mismatch). All four combinations were used in both a SR sentence and the corresponding OR sentence. The subject NP of the relative clause verb was always a proper noun (e.g. *David*).

*Semantic congruency experiment.* In the semantic congruency experiment, the relative clause verb took a direct object complement. Half of the stimulus sentences were semantically and grammatically matching OR–SR sentence pairs. The agent NP always referred to a human (e.g. *flickan *‘the-girl’) and the object of the relative clause verb was an inanimate NP (e.g. *bröd *‘bread’). For each matching sentence, a semantically incongruent version was created by replacing the verb of the relative clause so that the preposed object NP became an implausible argument of the relative clause verb (e.g. *bakar bröd *‘bake bread’ was replaced by *läser bröd *‘reads bread’). The sentences of the incongruent condition were grammatically well-formed, and all the verbs used in the relative clauses were transitive. In addition, each verb appeared both with a semantically congruent and incongruent object within the same lexical sentence frame (*läser bröd *‘reads bread’ in one set was counterbalanced by the well-formed *läser böcker* ‘reads books’ in another set).

### Procedure

The experimental procedure was the same in both the grammatical congruency and the semantic congruency experiment. Stimulus presentation and the recording of response times (RTs) were controlled through a PC running E-prime software. Following a practice block of 6 items, the experimental sentences were presented in pseudo-randomized order, distributed over 6 blocks. Presentation rates were randomized within a block. Sentences were shown word by word, in white font against a black background at the center of a computer screen. The stimulus onset asynchrony (SOA) including an interstimulus interval of 50 ms was 350 ms at fast presentation rate, 675 ms at medium rate and 1050 ms at slow rate. The participants were requested to respond to the final word of each sentence (marked by underlining) as quickly as possible. In the grammatical congruency experiment, the task was to determine if the last word was correct or incorrect by pressing one of two keys (1 = Correct, 2 = Incorrect). In the semantic congruency experiment, the participants were instructed to judge the last word as “OK” (1) or “strange” (2) based on the meaning of the sentence. Individual RTs were recorded for each sentence-final word.

### Data Analysis

*Accuracy.* Accuracy rate data obtained for the sentence-final judgment task was subjected to repeated measures ANOVAs. In the grammatical congruency experiment, the within-subjects factors were Syntax (levels: OR, subject relative), Rate (levels: slow, medium, fast) and Grammaticality (levels: grammatical, ungrammatical), and in the semantic congruency experiment, the factors were Syntax, Rate and Congruency (levels: congruent, incongruent).

**Fig. 1:**
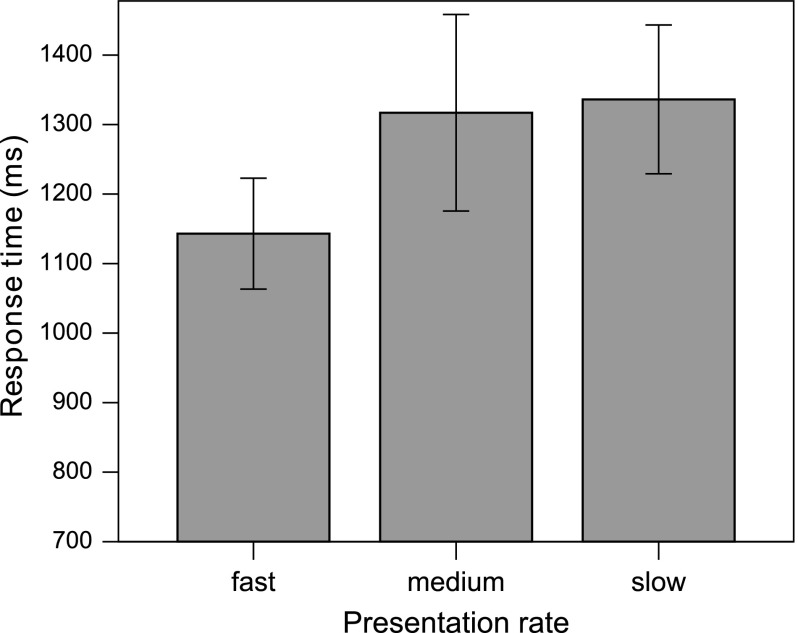
Mean response times for mismatching adjectives in object relative sentences at three presentation rates in the grammatical congruency experiment. Error bars show the standard error of the mean. The distance between the noun phrase and the adjective was 1.76 s at fast rate of presentation, 3.38 s at medium rate and 5.25 s at slow rate

**Fig. 2:**
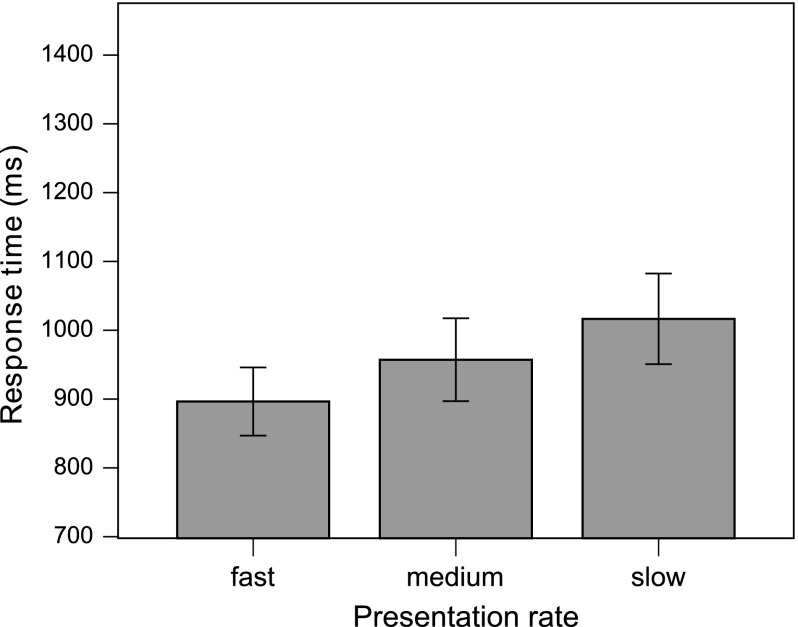
Mean response times for incongruent verbs in object relative sentences at three presentation rates in the semantic congruency experiment. Error bars show the standard error of the mean. The distance between the preposed object noun phrase and the verb was 1.76 s at fast rate of presentation, 3.38 s at medium rate and 5.25 s at slow rate

*Response times.* Only trials that received correct responses were included in the analysis of the response time data. RTs were log-transformed to approximate a normal distribution, and the original values in milliseconds are shown in parentheses in the results (see also Figs. 1, 2). Repeated measures ANOVAs were performed by subjects (*F*1) and by items (*F*2). In the grammatical congruency experiment, the factors were Syntax (levels: object relative, subject relative), Rate (levels: slow, medium, fast) and Grammaticality (levels: grammatical, ungrammatical), and in the semantic congruency experiment, the factors were Syntax (levels: OR, subject relative), Rate (levels: slow, medium, fast) and Congruency (levels: congruent, incongruent). Dependent measures were the RTs for sentence-final words, calculated from the presentation of the word. When conducting pairwise comparisons between the levels of Rate, the Bonferroni procedure was used to adjust the probability level.

## Results

### Grammatical Congruency Experiment

*Accuracy.* Overall accuracy on the judgment task was high, $M = 94.75\,\%$, *SD * = 3.52 %. Participants generally responded more accurately to SRs, $M = 97.91\,\%$, *SD* = 4.10 %, than to ORs, $M = 91.49\,\%$, *SD* = 9.84 %, resulting in a main effect of Syntax ($F(1, 26) = 44.823, p < .001$).

*Response times.* In the ANOVAs conducted on the response time data, presentation rate had an effect on the RTs of ungrammatical OR sentences, where fast presentation yielded enhanced processing speed at fast rate as compared to both medium and slow (Fig. 1). The global analysis found a main effect of Syntax ($F1(1, 27) = 16.05, p < .001; F2(1, 19) = 6.17, p = .022)$, which was modified by a marginal Syntax $\times$ Rate $\times$ Grammaticality interaction ($F1(2, 54) = 3.111, p = .053; F2(2, 38) = 2.808, p = .073$). The analysis was, therefore, broken down by the factor Grammaticality, resulting in a Syntax $\times$ Rate interaction for ungrammatical sentences ($F1(2, 54) = 10.082, p < .001; F2(2, 38) = 5.308, p = .009$), but not for grammatical sentences. Further analysis found an effect of Rate for ORs ($F1(2, 54) = 9.107, p < .001; F2(2, 38) = 4.857, p = .013$), reflecting the fact that RTs to ORs with ungrammatical final words were significantly shorter when presentation rate was fast ($M = 1143$ ms), relative to both medium ($M = 1317$ ms, $p = .018$) and slow rate ($M = 1336$ ms, $p= .001$). For SR-ungrammatical, there was no significant difference between slow ($M= 1167$ ms), medium ($M = 1240$ ms) and fast ($M = 1251$ ms) presentation rate.

### Semantic Congruency Experiment

*Accuracy.* Average accuracy for the twelve experimental conditions was high, 98 % (*SD*  = 0.75 %). As in the grammatical congruency experiment, the ANOVA revealed only an effect of Syntax ($F(1, 24) = 9.717, p = .004$), which was due to significantly higher accuracy scores for SRs, $M= 98.51\,\%$, *SD* = 2.66 %, relative to ORs, $M = 97.5\,\%$, *SD* = 3.98 %.

*Response times.* RTs showed an effect of presentation rate on incongruent OR sentences. Again, participants responded quicker at the fast presentation rate, but the effect was more gradual than that found in the grammatical congruency experiment (Fig. 2). Thus, a repeated measures ANOVA carried out on the response time data found an effect of Syntax ($F1(1, 27) = 9.02, p = .006; F2(1, 19) = 14.76, p = .001$), modified by a Syntax $\times$ Rate $\times$ Congruency interaction in the subjects analysis ($F1(2, 54) = 3.845, p = .027; F2(2, 38) = 1.475, p = .242$). Separate tests for each level of the Congruency factor revealed a Syntax $\times$ Rate interaction only for incongruent sentences ($F1(2, 54) = 8.268, p = .001; F2(2, 38) = 3.511, p = .040$), where the effect of Rate was significant for OR sentences ($F1(2, 54) = 11.158, p < .001; F2(2, 38) = 10.308, p < .001$). Pairwise contrasts indicated that RTs for OR-incongruent sentences at fast presentation rate ($M= 899$ ms) were significantly shorter than at slow rate ($M = 1019$ ms, $p < .001$), and, in the items analysis, marginally shorter than at medium rate ($M = 960$  ms, $p= .081$). For SR-incongruent sentences, no significant difference between slow ($M = 898$ ms), medium ($M = 886$ ms) and fast ($M = 881$ ms) rate was found.

## Discussion

The results indicate that time constraints affect working memory load during sentence processing. In both experiments, we varied the rate with which participants read OR and SR sentences in order to examine the processing of linguistic dependencies below and beyond the proposed 3 s time-window. Response times were recorded for the second member of the dependency, which was a grammatically congruent versus incongruent adjective in the grammatical congruency experiment and a semantically congruent versus incongruent word in the semantic congruency experiment. In OR sentences where the temporal interval separating the same words was either shorter (fast rate) or longer (medium and slow rate) than the assumed time-window, both grammatical and semantic congruency judgments showed an effect of presentation rate in incongruent sentences. Results from the grammatical and the semantic tasks revealed different tendencies: whereas processing time at sentence final verbs semantically incongruent with preceding extraposed objects increased gradually from fast to slow presentation rate, response latencies for sentence final adjectives with mismatching grammatical agreement features were similar at slow and medium rate and significantly reduced at fast rate. Presentation rate generally did not influence the processing of ungrammatical or incongruent SR sentences, where the dependent words always appeared within the hypothesized time-window.

Results concerning the integration of grammatical features are clearly indicative of distinct processes below and beyond 3 s. This pattern was observed in the ungrammatical condition, where response latencies were assumed to reflect the time it took to process the grammatical agreement dependency. Encountering the grammatically incorrect sentence final adjective could be thought to trigger a search in memory for a constituent with matching number features. This process seems to have been rapidly resolved when the temporal distance between the preceding NP and the adjective was only 1750  ms. At this fast processing rate, the previously presented member of the dependency could be easily found if the grammatical form of the NP was still activated in working memory. The sharp dividing line separating the shortest interval from the other two presentation rates suggests that the grammatical agreement mismatch was established based on a different, more time-consuming process at distance greater than 3 s. Previous results have shown reduced contribution of grammatical forms to the agreement processing mechanism beyond the limits of the hypothesized time-window (Roll et al. 2013). From this perspective, the observed increase in response latencies associated with intervals of 3375 and 5250 ms between the NP in the main clause and the sentence final adjective could indicate that the grammatical form of the NP had already faded and the retrieval of the earlier member of the dependency required an extended search in sentence semantic (propositional) representations.

In the semantic congruency experiment, response time patterns for sentence final unexpected verbs did not display a qualitative change when the temporal delay between the verb and a preceding extraposed object exceeded 3 s. This result is in line with the assumption that the 2–3 s long time limit is associated with the decay of grammatical information: resolving the semantic fit between an incongruous verb and its object would require access to the semantic representations of the dependent words and would not crucially rely on the grammatical form of the NP. Since processing time showed a steady increase as the distance between the verb and its argument increased, it is possible that readers found it more difficult to establish the semantic congruency due to a gradual decline in the activation of word semantic features when transition to more sentence (propositional) and discourse semantic representations could be expected. At the same time, the loss of form-based information after 2–3 s could also have contributed to the observed steady increase in response time pattern. Having the grammatical features of the object NP, such as its syntactic category activated in working memory at short temporal distance might have facilitated the structural association of the extraposed argument with the object position of the final verb, and, in turn, the subsequent establishment of the semantic mismatch between the two constituents. From this perspective, the gradual increase in response times could be explained as follows: at fast presentation rate, not only were the semantic features of the preposed object strongly activated at the final verb but also its associated syntactic information, leading to a rapid identification of the semantic mismatch. At medium presentation rate, the loss of syntactic detail could have slowed down the process of linking the preposed object to its verb, which possibly contributed to the observed increased response times. Finally, at the slowest presentation rate, an additional decline in the activation of word semantic information associated with the extraposed object further increased response latency.

No interaction between sentence structure and presentation rate was found for the congruent sentences, which suggests that readers were generally able to verify the correctness of the final word in these conditions without any extended memory search process. As previously discussed, sentence comprehension is assumed to involve the recoding of detailed form-based information into a sparser semantic representation. This type of representation extracted from the previously presented part of the sentence is presumably readily available upon processing each incoming word. Participants could be thought to have made their decision as soon as they encountered a target word that directly fit into this meaning representation, such as an adjective with a plural specification in case a proposition was made about plural entities, or a semantically congruent verb. If such a fit could not be established, as was the case in the incongruent conditions, test persons were presumably able to make their response decisions only after a backtracking process during which the match between the final target word and the previously presented member of the dependency was evaluated. Thus, response latencies varied in these conditions depending on whether the form of previously processed words had vanished and, in the semantic congruency experiment, on the degree to which word semantic representations declined over time.

Overall, the results indicate that temporal distance might influence the integration of linguistic information. Grammatical forms in working memory seem to decay within 2–3 s, constituting a short time-window for rapid form-based agreement resolution. Timing constraints on working memory, therefore, might contribute to the comprehension difficulty associated with OR sentences, in which linguistic dependencies may span over relatively long time-intervals.

## Footnotes

1 It should be noted that processing the semantic congruency also implies processing the syntactic dependency between the clefted constituent (*bröd *‘bread’) and its base position in the OR-structure. Nevertheless, unlike in the case of the grammatical congruency experiment, it is not the form of the previous NP object that provides crucial cues to detecting the mismatch in the semantic task. Therefore, the decline of grammatical information might contribute to an increase in processing time, but this would not be expected to affect the response latency pattern.
